# A case report of cervicothoracic penetrating injury with retention of foreign body

**DOI:** 10.1186/s12893-021-01234-y

**Published:** 2021-05-03

**Authors:** Yang Hui, Xinxin Yang, Dengdian Ma, Mengwei Yao, Xinying Liu, Yunbing Dai, Qinyuan Huang, Tao Liu, Jing Xu, Xiaoyu Li

**Affiliations:** 1 School of Clinical Medicine, Jining Medical University, Jining, 272029 Shandong China; 2 Departments of Otolaryngology-Head and Neck Surgery, Affiliated Hospital of Jining Medical University, Jining, 272029 Shandong China; 3 Department of Gynecology, Affiliated Hospital of Jining Medical University, Jining, 272029 Shandong China

**Keywords:** Chest trauma, Neck trauma, Penetrating trauma, Foreign body

## Abstract

**Background:**

Cervicothoracic penetrating injury, considered to be relatively rare, has a complicated mechanism that is difficult to treat. In this report, a special case of cervicothoracic injury caused by foreign body penetration was elucidated. In this case, the injury location and the involved foreign body were exceptionally particular, which induced a challenging process of diagnosis and treatment.

**Case presentation:**

A male patient suffered from a serious injury caused by a thick branch that pierced through his neck in a traffic accident between an electric car and a tricycle carrying wood. There were also local injuries in the left scapular region. After an emergency multidisciplinary consultation, the patient was diagnosed and subsequently treated with vascular exploration and repair (common carotid artery), intrathoracic foreign body extraction, chest exploration, debridement, and suture. After surgery, he was transferred to the emergency intensive care unit for anticoagulation and anti-infection treatment. Finally, after the improvement of his physical condition, the patient was transferred to the general ward for further treatment and was successfully discharged from the hospital. Once discharged, the patient lived a normal life, free from sequelae or complications.

**Conclusion:**

It may be an extremely daunting task to cure cervicothoracic penetrating injury due to its rare occurrence in clinical practice. Different from the previous cervicothoracic traumas, the injury location in this case is exceedingly particular. In general, the common cervicothoracic trauma is associated with damage to the trachea, esophagus, throat, and other structures, easily resulting in dyspnea, which, however, does not occur in this case. The insertion position of foreign body is exceptionally particular as it does not pierce the common carotid artery but poses compression on it, which induces ischemia. It is essential for the successful treatment that the treatment plan is formulated via the detailed imaging examination and careful multidisciplinary consultation.

## Background

Penetrating neck injuries are the rarest form among all traumatic injuries at our department [1]. Neck trauma is a significant surgical emergency faced by ENT surgeons in their clinical practice. Timely presentation to the referral center and proper multidisciplinary management approach play a pivotal role in the wound healing and prevention of such serious complications as shock, sepsis, laryngeal stenosis, and fistula formation [2].

## Case presentation

A 41-year-old male patient was admitted to Affiliated Hospital of Jining Medical University with a "stab wound to the neck and chest caused by a branch" that occurred in the evening on November 1, 2019, 5 h before the patient was admitted to our hospital. The patient drove an electric car and crashed into a tricycle carrying wood, which resulted in a branch stabbing him into the neck without causing coma, disturbance of consciousness, chest tightness, palpitation, dyspnea, or dizziness. For that reason, he was admitted to Yanzhou People’s Hospital after the traffic accident and was recommended to be transferred to our hospital for further treatment. He was admitted to the hospital initially with "cervicothoracic branch penetrating injury" after an emergency examination. Subsequently, the patient was sent to the operating room through a green channel. He had a history of "bilateral congenital cataract" and decreased vision, but no history of hypertension, diabetes, coronary heart disease, and similar diseases.

Physical examination at T36.5 ℃ P100/min R20/min BP164/98 mmHg showed irregular laceration and bleeding in the lower jaw. In the middle of the neck, the branch with 5 cm in length pierced the left scapular region, which caused a shift of the trachea and local elevation of the left scapular region, accompanied with a shortness of breath but without rales and arrhythmia in both lungs. Abdominal weakness was observed, free from tenderness and rebound pain. The fluctuation of the left radial artery was weak, and the sensation in the ulnar nerve control area of the hand was decreased.

**Fig. 1:**
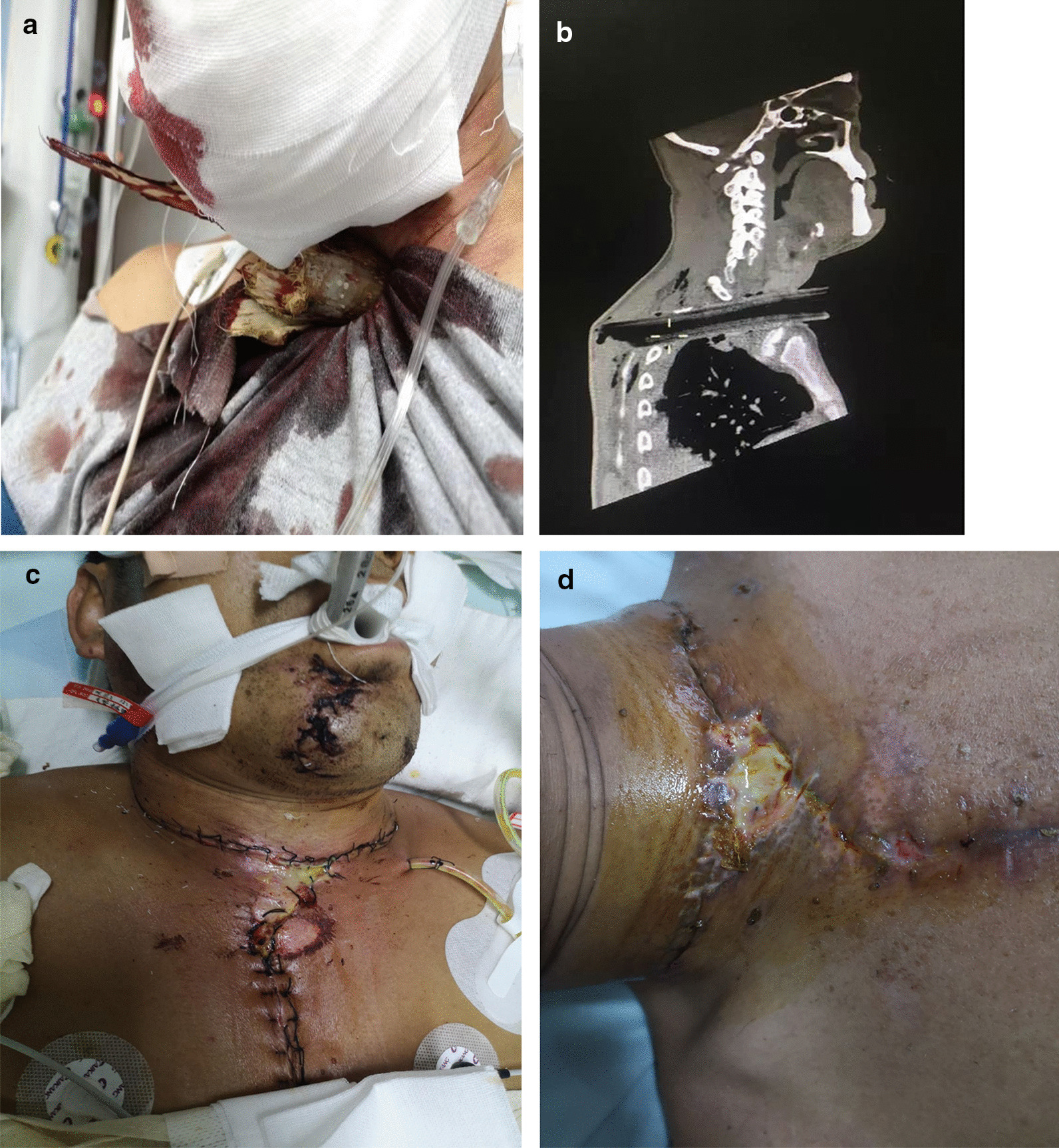
**a** The patient was stabbed by a thick branch penetrating from the neck to the chest and back. **b** The CT imaging data of the patient. **c** The dressing was changed, and the wound condition was observed (2019.11.07). **d** The wound condition of the patient after the suture was removed (2019.11.21)

The auxiliary examination was performed on November 1, 2019. The dual-source CT cervical angiography emergency report revealed the following facts: cervical anterior-left lung tip-neck back penetrating injury with retention of foreign body, injury to surrounding soft tissue, gas accumulation, proximal left common carotid artery, and left subclavian artery proximal injury (our hospital, Piece number CT201911011762). Admission diagnosis included cervicothoracic branch puncture, maxillofacial skin and soft tissue contusion and laceration, cervicothoracic foreign body retention, cervicothoracic vascular injury, brachial plexus injury, and congenital cataract. Due to the fact that the patient suffered from a penetrating injury to the neck and chest, the patient’s condition was further complicated by penetrating injuries to his neck and chest, and it required an extremely challenging operation that could hardly be performed by a single department. Consequently, the hospital immediately activated Multiple Disciplinary Team (MDT) to summon experts urgently from departments of otorhinolaryngology, head and neck surgery, cardiac surgery, thoracic surgery, vascular surgery, and orthopedic trauma to conduct multidisciplinary consultation and formulate treatment plans. On account of the cooperation with a common effort among imaging department, laboratory department, and emergency department, necessary examination results were provided in a timely manner, which contributed to the revealing of satisfactory conditions of the patient. According to the results and the discussion of experts, the main difficulties of the operation contained these three aspects. Firstly, as for the puncture wound in the patient’s neck and chest, there are many important blood vessels, nerves, and other structures on the neck. Besides, the foreign body has barks, irregular shapes, and rough surfaces. Consequently, it is necessary to fully expose the wound, with the aim of avoiding damage to the surrounding tissue Secondly, there are greater possibilities of the occurrence of vascular injury in cervical puncture injury, the injury to the common carotid artery, subclavian artery, and other important blood vessels adjacent in particular. As even the minimal error may result in massive bleeding, the focus of this operation is the treatment and protection of blood vessels. Thirdly, since the foreign body was still lodged in the neck, mediastinal infection, atelectasis, tracheoesophageal fistula, chylothorax, and other complications may occur once it was pulled out. The multidisciplinary team reached the following conclusions. First of all, the operating room and anesthesiology department was informed to make full preparation before the operation, where special attention was given to posture protection in order to avoid secondary injury. Besides, the cardiopulmonary bypass team was informed to be in place with a view to avoiding massive bleeding. Moreover, experts from all departments were all in place in the operating room. Furthermore, we communicated with the family members one more time before the operation. Finally, all experts agreed that they had made full preparations to perform soft tissue exploration, repair, and surgical treatment of foreign bodies (Fig. 1).

After entering the operating room, all tasks were completed before the operation. After the anaesthetic took effects, the otolaryngologist firstly performed vascular exploration and repair (common carotid artery), intrathoracic foreign body extraction, thoracotomy, and debridement suture. In addition, the otolaryngologist made a low-collar large incision that allowed the wound could be exposed layer by layer. The foreign body was located at the inner edge of the sternocleidomastoid muscle, where it punctured the common carotid sheath and penetrated between the common carotid artery and the internal jugular vein. The pulsation of the common carotid artery was not involved. The left thyroid lob was slightly contused, and a further lateral exploration was necessary. Chylous fistula also appeared. Due to the obvious thickening of the external jugular vein and transverse jugular vein, the operation was further extended by suspending the internal jugular vein with a rubber hose, and the foreign body was found to be immobile. Due to the fact that the foreign body could not be removed from the neck, the second group of cardiac surgeons tried to split the sternum and further expand the surgical field of vision. As a result, the left common carotid artery was exposed, and there was no pulsation at the distal end. The foreign body posed compression on the proximal end of the left common carotid artery and local adventitia hematoma. Median thoracotomy was performed, and the left common carotid artery near the cardiac end outside the pericardium was dissociated. The foreign body, with about 5 cm in diameter and about 30 cm in length, was removed. There was no blood spurting through the foramen and no occurrence of rupture in the left common carotid artery. There was no recovery of the distal pulsation of the left common carotid artery. Provided with 1 mg/kg heparin, the left common carotid artery was occluded by the longitudinal incision of the left common carotid artery. The diseased vessels were resected, and the 8 mm artificial vessels were anastomosed end-to-end, followed by the suture with 5-0prolene. After full exhaust, the distal blood flow was restored. The bleeding was thoroughly stopped, the median incision was closed layer by layer, and a drainage tube was placed. Then, the stomatologist performed the removal and debridement of the skin and subcutaneous necrotic tissue of the lip. Finally, the wound was repaired by an otolaryngologist. On the ground that the foreign body was a branch with a rough surface, thoracic surgery followed otorhinolaryngology, cardiac surgery, and oral surgery. The observation hole with 1 cm in diameter was introduced at the seventh intercostal space of the left midaxillary line. Besides, the length of the fourth intercostal space of the axillary midline, which was taken as the operation hole, was about 3 cm. There was a large amount of blood fluid in the chest cavity, with the amount about 500 ml. It could be seen that contusions existed at the tip of the lung, and the foreign body located in the posterior wall of the chest, where it was embedded into the intercostal muscle. It could be seen that turbid fluid exudation was present at the top of the chest. The foreign body embedded in the chest wall was not removed until the incision was enlarged to 10 cm. Multiple ligations of the thoracic duct were observed under the aortic arch. At the end of the operation, there was no air leakage in both lungs, and the chest was repeatedly washed with warm saline. There was no lymph exudation in the thoracic cavity. At the end of the operation, one retention tube was placed in the observation hole. The operation was completed successfully, with the bleeding about 1500 ml, the infusion about 5000 ml, the urine volume 2800 ml, the transfusion 6U, the plasma 600 ml, and the blood transfusion going smoothly, and there were no obvious abnormalities occurring to the patient. On November 2, 2019 after the operation, the patient was transferred to the emergency intensive care unit.

After the operation, the patient received comprehensive treatment in the emergency intensive care unit, where he was given oral tracheal intubation and ventilator-assisted ventilation. With the consideration that the patient suffered severe trauma, severe stress reaction, and contaminated wound, the postoperative treatment included anticoagulant and anti-infective therapy. Treated in the emergency intensive care unit, the patient was provided with piperacillin tamartan, levofloxacin, and tinitramine for anti-infection, with reduced glutathione to protect the liver, with vitamin C to improve capillary permeability, with ulinastatin to reduce the inflammatory reaction, with mannitol to reduce tissue edema, with acetylcysteine to reduce phlegm, with papaverine to improve microcirculation, with pantoprazole to inhibit acid and protect the stomach. Dressings were also changed regularly to keep clean. After entering the intensive care unit, the patient had a recurrent fever. Piperacillin tazobactam, levofloxacin, and tinidazole were stopped on November 07, and imipenem cilastatin, linezolid, and minocycline were provided as anti-infective treatment. With the gradual improvement of the patient's condition, he was transferred to the orthopedic trauma ward on November 14, 2019 and treated with imipenem cilastatin, linezolid, and minocycline for anti-infection, anticoagulation, and improvement of microcirculation.

After being evaluated on his condition, the patient was discharged from the hospital on November 25, 2019. He came back to the hospital for reexamination 1 week after discharge and was followed up by telephone at 1 month, 3 months, and 5 months after discharge. The patient was in good condition, free from complications and sequelae.

## Discussion and conclusions

Penetrating neck injury accounts for 5–10% of all trauma cases [3]. By virtue of mortality rates as high as 10%, it is necessary for clinicians to be familiar with management principles [4], which, however, is proven to be difficult due to the fact that there are no international consensus guidelines. Fortunately, recent improvements in imaging modalities have altered the ways in which such cases are being approached [5]. No other important vital structure in the human body occupies a limited space such as the neck. Major vascular, enteric, and neurologic conduits span the short gap between the head and the torso, including the spinal cord, the esophagus, and the carotid and vertebral arteries, which supply blood flow to the brain. Unrecognized neck injuries can be easily fatal, either on account of rapid exsanguination or airway occlusion and asphyxiation. The carotid arteries carry a significant proportion of the total cardiac output, and they may either profusely bleed or be obstructed once being injured. The resultant sudden fall in cerebral perfusion may produce focal or global neurologic deficits that are frequently irreversible if not fatal [6]. The main cause of death is vascular injury and massive bleeding [3]. In cases of major hemorrhage, the initial goal is to identify and control bleeding vessels. Defects of the common carotid, internal carotid, or external carotid arteries should also be repaired if possible. Larger defects may require ligation, primary re-anastomosis, or grafting with the autologous vein, polyester (Dacron), or polytetrafluorethylene (PTFE) material. If the patient is hemodynamically stable, it is worth trying to perform the endovascular repair by specialist neuro-interventional radiologists or vascular surgeons. Much attention shall be paid when selecting an operative approach. The ideal incision should allow adequate exposure of the injury area with the ability to extend the incision if necessary [7]. As for penetrating trauma, immediate life-saving measures, rapid transfer to a tertiary trauma center, appropriate intensive care, a series of reassessed targeted examinations in the emergency room, timely diagnosis, and surgical intervention by multidisciplinary teams are necessary procedures [8]. It is also crucial for the successful treatment of the patient to formulate diagnosis and treatment plans in detail before, during, and after operation. For example, after being admitted to the hospital, the vital signs of the patient were still stable. As per findings in relevant examinations, we immediately held a multidisciplinary consultation to formulate the operation plan, set up a medical first aid team, contact the operating room at the same time, and make all preoperative preparations, taking into account how to expose and remove foreign bodies and how to prevent sequelae and complications. During the operation, it was necessary to firstly consider adequate exposure of the surgical field in order to explore whether there was any injury to the important blood vessels of the neck, such as the common carotid artery and the subclavian artery. Besides, it was also necessary for cardiac surgeons to explore the heart and large vessels to ensure that the life-threatening bleeding could be stopped eventually. During the operation, it was found that the foreign body penetrated between the common carotid artery and the internal jugular vein and punctured the carotid sheath. However, the pulsation of the common carotid artery was not involved, even after the foreign body was pulled out. Therefore, it was necessary to consider the possible consequences and complications of common carotid artery occlusion.

In 1872, Verneuil initially reported cases of carotid artery thrombosis and cerebral infarction after cervical trauma [9]. The mechanisms of cerebral infarction caused by post-traumatic carotid artery thrombosis can be explained as follows. The intimal injury in the neck is mainly caused by intimal tear and contusion, which in turn is caused by excessive tension, compression, flexion and torsion of the blood vessels under the action of external force. When the internal carotid artery passes through the bony structure such as the lateral mass of the axis or the transverse process of the third cervical vertebra, the above external forces may destroy the integrity of vascular intima, which would form a rough surface, matrix exposure, platelet adhesion, and mural thrombus. Large mural thrombus can occlude blood vessels and cause cerebral infarction; while small mural thrombus can constantly fall off, and the shedding embolus can drift to smaller blood vessels with blood flow, which would induce a cerebral embolism. The mechanism is carotid artery traumatic non-atherosclerotic artery-to-artery embolism [10]. The mortality rate of ligation of the common carotid artery and internal carotid artery, which needs to be repaired by blocking blood flow or bypass, has been reported to be 12%–50. Cerebral infarction caused by cerebral ischemia and thrombus occlusion are highly risky [11]. In this case, the incision of the common carotid artery revealed that the local intimal tear and distal inversion would induce the occlusion in the left common carotid artery. The diseased segment was resected and anastomosed with artificial blood vessels. Besides, it could be found that the blood flow recovered after completing suture. However, it is necessary to conduct further treatment and observations to learn whether therewould be sequelae after surgery. Moreover, thoracoscopic exploration was performed by thoracic surgery to remove the remaining foreign bodies and ligate the thoracic duct to repair the damaged lungs. According to relevant literature reports, indications for removal of residual foreign bodies from the chest include septicemia, migratory foreign bodies that may be embolized, lead poisoning or massive hemothorax, hemothorax clots, empyema, or packing that may occur during exploration for other reasons. Anticoagulation, anti-infection, mechanical ventilation, and reducing brain edema after the operation also exert significant impacts on the treatment for patients [12].

To sum up, the successful treatment for this patient provides a valuable reference for similar cases. First of all, it is necessary to keep the respiratory tract unobstructed. Before the precise determination of diagnosis and treatment, it plays a decisive role to keep foreign bodies in place and fix it properly in order to prevent extraction and wobbling. In addition, the patient shall be sent directly to a hospital for the treatment as soon as possible. On the ground that the branch penetrates between the common carotid artery and the internal jugular vein and it punctures the common carotid artery, the intima would flip and occlusion would occur in the common carotid artery. Fortunately, as the trachea, esophagus, and throat are not damaged, which can increase the patient's chance of survival, the patient can obtain the opportunity to be sent to the hospital, which provides valuable time for rescue intervention.

Meanwhile, both the timely multidisciplinary consultation and the opening of the green channel exert significant impacts on the treatment in this case. The emergency green channel is an effective mechanism used for the treatment of critically ill patients. The emergency departments of national hospitals have reached a consensus on providing effective measures for critically ill patients. The emergency green channel makes the rescue process possible for emergency and serious cases. Furthermore, it also provides a timely diagnosis and treatment process that are indispensable for saving patients' lives, thus improving the success rate of rescuing patients and reducing medical risks. After the patient is admitted to the hospital, the green channel is available, and the medical staff conducts a preliminary evaluation and rescue management. Emergency nurses have achieved significant impacts on communication, cooperation, and guidance [13].

Detailed preoperative imaging examination is the first step in cervicothoracic injury, because it can clearly indicate the relationship between the penetration path of foreign body and the surrounding tissue, which contributes to providing better guidance for the formulation of a surgical plan. Although penetrating neck trauma is relatively rare, clinicians should have a thorough understanding of the area’s anatomy and be familiar with the preliminary steps for dealing with these cases. Computed tomographic angiography (CTA) is the first choice for imaging examination of hemodynamically stable patients with cervical penetrating injury. Computed tomography (CT) and 3D reconstruction images can also provide good guidance for clinicians to rule out macrovascular injury. Imaging examination should also be performed after the operation to confirm that there are no foreign body residues left in the wound [3]. The extraction of the foreign body should be carried out under direct vision, which is essential for the successful operation. The deep part of the foreign body shall be exposed adequately throughout the operation, and the surrounding nerves and blood vessels shall be protected, after which the foreign body should be removed gently and slowly as far as possible, on the ground that injury can be further aggravated by the excessive movement of the foreign body [4]. After the removal of the foreign body, it is necessary to check whether the foreign body has been fully removed. For the patient in this case, video-assisted thoracoscopic exploration is carried out after the removal of the foreign body, after which the residues of the foreign body have been successfully removed. Careful exploration, layer by layer exposure, protection and treatment of blood vessels, postoperative anticoagulation, anti-infective treatment, and monitoring of vital signs provide a guarantee for the final recovery and discharge of patients. The daunting task can be accomplished due to careful deployment and implementation of every step, as well as the precision and speed of multi-disciplinary cooperation in Affiliated Hospital of Jining Medical University, which is worthy of our continuous promotion and advancement.

## Data Availability

Data sharing is not applicable to this article, as no datasets have been generated or analyzed during the current study.

## Abbreviations

PTFE: Polytetrafluorethylene
ENT: (Ear–nose–throat) department
CT: Computed tomography
MDT: Multiple disciplinary team
ECG: Electrocardiogram
CTA: Computed tomographic angiography
